# Supplementary material for: Learning curve and surgical outcome of robotic assisted colorectal surgery with ERAS program
Source: Sci Rep. 2022 Nov 29;12:20566. doi: 10.1038/s41598-022-24665-w (PMC9709162; doi:10.1038/s41598-022-24665-w)
Supplement: Supplementary file 2 — Supplementary Information 2. [file 41598_2022_24665_MOESM2_ESM.zip › RAL Raw data-Ñ[▒K/128/OP N_ñw╝╨░O▒Kñσ.pdf]

W52 / W52

臺中榮民總醫院
手術室護理記錄

[redacted]

基本資料

- 室別： 開刀房-17室，手術科別： CRS，手術類別： 常規，日期： 2021/11/20
- 進入時間： 0745 ； 麻醉開始時間： 0805 ； Time Out時間： 0850 ； 劃刀時間： 0851 ； 手術結束時間： 1615 送出時間： 1620 ；
- 麻醉方式： GE

  診斷： Malignant tumor of rectum

  手術名稱： Robot Assisted Low Anterior Resection

  手術局部用藥： nil

  術前預防性抗生素：
  - 抗生素名稱一： Veterin 1GM IVD/由麻醉護理人員給予
  - 時間： 0835
  - 上次簽章: 歐真勤

  ，追加預防性抗生素：
  - 抗生素名稱一： Veterin 1GM IVD/由麻醉護理人員給予
  - 時間： 1320
  - 上次簽章: 歐真勤
- 手術醫師： 蔣鋒帆，陳瑨夫，余泓均
- 麻醉醫師： VS 謝昀叡
-
  - 刷手護士A ： 童歐文，時間： 0745，刷手護士B ： 廖子晴，時間： 0745
  - 流動護士A ： 歐真勤，時間： 0745，接替流動一： 廖子晴，時間： 1130，接替流動二： 歐真勤，時間： 1200

皮膚狀況

A-V Shunt： 無

術前： 完整

記錄者： 上次簽章: 歐真勤

術後： 完整

記錄者： 歐真勤

手術準備

手術臥式 單一手術臥式

-
  - 手術臥式： 截石臥式
  - 使用附件： 溫毯 38 ℃，頭圈，腳架
  - 皮膚準備： 10%Aq Betadine，Hibiscrub，2% Chlorhexidine
  - 電燒： 使用電燒: 電極片位置： 其它： 導電軟墊，雙極電燒，記錄者： 上次簽章: 歐真勤

止血帶T： 無

植入物

無

標本送檢

- 細菌培養 無
- 細胞學 無
- 冰凍切片 部位: 1. peritoneum，次數： 1
- 病理標本

  組織名稱一： rectum

  ,

  組織名稱二： IMA root

  ,

  組織名稱三： distal cut-end

- 截肢 無

標本交付 標本： 3 袋，標本： 0 盒，標本： 0 瓶，細胞學： 0 管，Culture Tube： 0 管，被交付醫師： CR 陳璿夫，

- 記錄者： 上次簽章: 歐真勤
- 時間： 1600

傷口術後敷料

傷口敷料： 傷口敷料： 無

- 請於圖上標示部位：

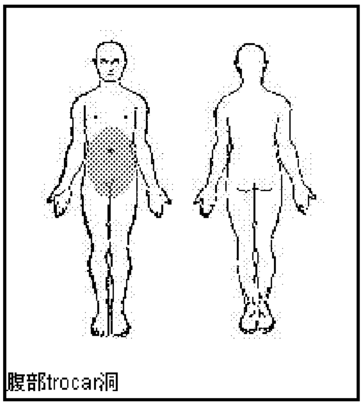

- 傷口敷料： mepore

導管

導管	天數	導管資訊	插入日期	插入時間	更換日期	拔除日期	拔除時間	拔除原因	預計拔除/更換	預計拔除/更換日期
Foley(1)	1	號碼:16 Fr.; 部位:膀胱;	2021/11/20	0900						
CVC(1)	1	號碼:8 Fr; 深度:15cm; 部位:jugular (右); 象限:RU;	2021/11/20	0835						
A-line (1)	1	號碼:20 Ga; 部位:左手;	2021/11/20	0825						
Double-J(1)	1	號碼:6 Fr.; 深度:26cm; 部位:輸尿管; 象限:LU;	2021/11/20	0900						

用物計數

- 手術中出血量： 50 c.c.
- 手術中尿量： 550 c.c.
-
  - 其它： 無

用物計數：

- 部位一： 尿道，1.器械： 正確，2.縫針： 未使用，3.刀片： 未使用，4.紗布： 使用白紗，5.腹部墊： 未使用，6.紗球： 未使用，7.PEANUTS： 未使用，8.紗條： 未使用，9.絲帶： 未使用，10.針頭： 未使用，11.Vessel loop： 未使用，12.Boots： 未使用，13.Jelco： 未使用，14.棉條： 未使用，
  - 第一次：
    - 刷手護士 ： 廖子晴
    - 流動護士： 上次簽章: 歐真勤
    - 時間： 0905

  ,
  - 第二次：
    - 刷手護士 ： 廖子晴
    - 流動護士： 上次簽章: 歐真勤
    - 時間： 0910

  ,
  - 第三次：
    - 流動護士：
- 部位二： 腹部，1.器械： 正確，2.縫針： 正確，3.刀片： 正確，4.紗布： 正確，5.腹部墊： 未使用，6.紗球： 未使用，7.PEANUTS： 未使用，8.紗條： 未使用，9.絲帶： 正確，10.針頭： 未使用，11.Vessel loop： 未使用，12.Boots： 未使用，13.Jelco： 未使用，14.棉條： 未使用，

- 第一次：
  - 刷手護士 ： 童歐文
  - 流動護士： 上次簽章: 歐真勤
  - 時間： 1445

,
- 第二次：
  - 刷手護士 ： 童歐文
  - 流動護士： 上次簽章: 歐真勤
  - 時間： 1500

,
- 第三次：
  - 刷手護士 ： 童歐文
  - 流動護士： 上次簽章: 歐真勤
  - 時間： 1510

-
  - 第一次：
    - 流動護士：

  ,
  - 第二次：
    - 流動護士：

  ,
  - 第三次：
    - 流動護士：

備註

備註： 30度達文西:NO1
gul150012111193631
guc150012111193627

- 記錄者： 上次簽章: 歐真勤
- 時間： 0805

填寫護理人員 歐真勤

正式摘要

W52 / W52

doctorRank:B
